# The Relationship Between Children’s Diet and Risk Factors for Cardiovascular Disease

**DOI:** 10.3390/nu18010166

**Published:** 2026-01-04

**Authors:** Claire Butorac, Vadin Bruot, Zane Johnson, Sibylle Kranz

**Affiliations:** 1 Department of Kinesiology, University of Virginia, Charlottesville, VA 22903, USA or claire.butorac@utah.edu (C.B.); nmj8yr@virginia.edu (V.B.); mtf9rb@virginia.edu (Z.J.); 2 Department of Nutrition and Integrative Physiology, University of Utah, Salt Lake City, UT 84112, USA; 3 Department of Public Health Sciences, School of Medicine, University of Virginia, Charlottesville, VA 22903, USA

**Keywords:** pediatric nutrition, disease prevention, chronic diseases, diet interventions

## Abstract

**Background/Objectives** The number of children with cardiovascular disease (CVD) risk factors is increasing in the United States. This review summarizes the current knowledge on the relationship between children’s diets and CVD risk factors in children aged 2–18 years. **Methods**: A systematic literature review was conducted using Covidence (PROSPERO registration CRD42024604406) in the three databases PubMed Central, Web of Science, and Embase to include publications published in English between January 2014 and December 2024 that contained the outcome measures total cholesterol, LDL, HDL, triglycerides, and blood pressure. Two independent researchers conducted title, abstract, and full-text screenings; a tiebreaker was used to resolve any conflicts. Risk of bias was assessed using the quality assessment forms included in the Covidence software. **Results**: Eighty-five studies met the inclusion criteria, and the results were stratified by age group to organize results in a logical manner and increase transparency. Many studies have reported significant relationships, particularly with blood pressure and HDL, but others have found no statistically significant relationships. **Conclusions**: While a plethora of studies investigating the relationship between diet and CVD risk factors in children are available, the large heterogeneity between the diet factors, diet assessment, outcome measurement methodology, and outcome variable selection varied greatly, affecting the ability to arrive at conclusive results and recommendations. It would be beneficial to develop universally accepted research standards that can be applied to future studies to reduce ambiguity in the understanding of the effect of diet on CVD risk.

## 1. Introduction

For the past three decades, cardiovascular disease (CVD) has remained the most common cause of death in the United States and worldwide [1]. CVD risk is associated with modifiable lifestyle factors, including diet and physical activity [2]. Originally considered a chronic disease affecting only adults, CVD risk factors were long ignored in children, but recent data show that the prevalence of CVD risk in children is increasing [3] and that risk factors established during childhood might track through the lifecycle into adulthood [4].

Standard risk assessment variables for CVD are included in statements and position papers of medical groups and health authorities. The Scientific Statement published by the American Heart Association (AHA) defines a set of ideal CVD risk values for 6–19-year-olds as having a total cholesterol (TC) <170 mg/dL, blood pressure <90th percentile of systolic or diastolic pressure (mmHg), absence of dyslipidemia (elevated triglycerides (TG), low high-density lipoprotein cholesterol (HDL) <40, and high low-density lipoprotein (LDL)) [5]. The AHA also notes that changes in TGs and cholesterol are commonly observed during puberty and that establishing cut points for diagnosis is challenging at this age; thus, providers are encouraged to look for overall patterns of dyslipidemia [5]. The American Academy of Pediatrics recommends that children ages three years and older should undergo annual blood pressure checks; hypertension (HTN) is diagnosed based on age, height, and sex for children under 13 years old and adult cut points are applied to children 13+ (blood pressure goals less than the 90th percentile for children under 13 and less than 130/80 mm Hg for adolescents 13 and up). A recent study conducted in 2088 adolescents showed that 45.4% of boys and 37.4% of girls had significant modifiable lifestyle risk factors for CVD [6], including HTN (high systolic or diastolic blood pressure), high plasma TG, low HDL, and high LDL.

CVD risk factors are associated with overall diet quality [7] but also individual diet components, such as salt or sodium intake [8] and the consumption of processed foods [9], usual diet components such as salt or sodium intake [8] and consumption of processed foods [9] in adults. Research on the effect of diet on risk factors in children is sparse, but some studies have shown that diet can significantly increase CVD risk in children and adolescents [10,11,12,13] while certain other dietary patterns are associated with a lower risk, such as adherence to the Mediterranean diet [14,15]. Age is an important factor to consider. As discussed in detail by Balasundaram et al., human growth and development are highly individualized; however, physiological and psychological developmental stages can be addressed, on average, by age [16]. For instance, children of elementary school age (5–11 years old) likely experience periods of growth spurts, which affect circulating concentrations of nutrients and metabolites. A previously published review on the detection of dyslipidemia in children used the same age categories as those applied here [17]. One reason for these categories is that, while age can be used as a general determinant of pubertal development status, when measuring other factors such as genital development, it has been found that pubertal developmental status within the same age groups plays a large role in determining risk factors, such as blood pressure. A study by Li et al. found that later stages of puberty correlated to an increase in BP (*p* < 0.01) [18]. Based on this data, children who are in late stages of puberty may be closer to hypertensive values when compared to earlier stages of puberty. This could potentially lead to misinterpretation of data when measuring the effects of diet on BP, especially if the individual is in late-stage puberty over the duration of the study period and is thereby likely to have increased BP due to physiological changes. The mechanisms behind these trends are still not fully understood but are thought to be influenced by hormonal changes [19]. Along with BP, studies have found that lipid levels also shift throughout the stages of puberty [20]. Due to currently available data, the relationship between CVD risk factors and pubertal stage must be regarded as a confounding variable when evaluating the effect of diet on CVD risk factors.

This systematic review was conducted to review the current knowledge on the effect of diet on CVD risk factors in children and adolescents aged 2–18 years old.

## 2. Materials and Methods

To summarize current published information on the relationship between dietary intake and CVD risk factors in children aged 2–18 years, a systematic literature review was conducted using Covidence software (Covidence 2025) using PRISMA guidelines. After consultation with the University of Virginia’s health sciences librarian, a preliminary search term syntax was created and the review registered in PROSPERO (CRD42024604406) on 24 October 2024. A modification to this protocol was filed on 21 February 2025 to include cross sectional study designs, in addition to the originally stated randomized clinical trials and cohort studies. Based on the research question, population, interventions, comparators, outcomes, and study designs (PICOS) were defined as follows: The population included was children and adolescents aged 2–18 years. The interventions and exposure variables of interest and self-reported dietary intake were measured. The comparators were the baseline or post-intervention levels of adherence to a specific diet or eating practices, as defined by the authors to meet the restrictions of each diet or pattern of intake in the population of interest. The outcome variables included the physiological, BP, and biochemical measures of CVD risk as assessed by serum blood levels of LDL, HDL, TG, and TC.

Study types included were randomized controlled trials, prospective cohort studies, cross-sectional studies, and longitudinal studies. Only papers in English published between January 2014 and December 2024 were included. Due to the PICOS of this specific review, the search strategy was developed to prioritize the population, exposure variables, and outcome variables. Thus, the search syntax was based on publications in English; 2014–2024; clinical trial; cohort; cross-sectional; longitudinal study; subjects aged 2–18 years old; dietary intake measured; or reported CVD risk factors. The search was conducted using PubMed Central, Embase, and Web of Science databases. Since these databases have slightly different requirements for the search language, the search syntax was modified and implemented (see Table 1). Efforts to refine the search by narrowing the search terms were abandoned after pilot search outcome checks revealed that some valuable research reports might be excluded. However, the papers met the inclusion criteria. In total, four investigators conducted screenings.

**Figure 1:**
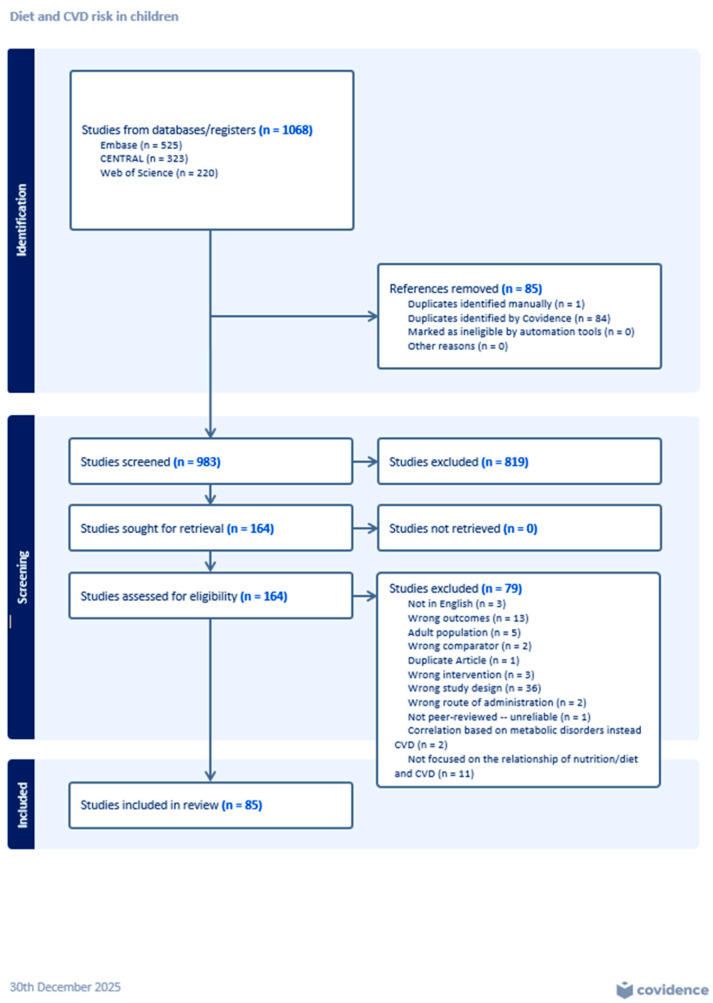
PRISMA chart.

For each database searched, Zotero’s reference manager collected and imported the articles and references into Covidence. Zotero removed some duplicate papers before uploading them to Covidence, lowering the number of duplicates shown in the Preferred Reporting Items for Systematic Reviews and Meta-Analysis (PRISMA) chart (Figure 1). Once in Covidence, article duplicates were removed by the system. Title and abstract screenings were conducted. Two researchers rated each paper, and a tiebreaker decided on conflicting decisions to minimize researcher bias. Publications included after title and abstract screening underwent full-text screening using the same method employed for the title and abstract screening.

After screening, 85 research reports were included in this summary of the systematic literature review. Information extracted for each study was the title, author, age of participants, type of study, outcome measures reported, preliminary findings, and overall conclusions drawn, which were compiled into an Excel sheet. The outcome measures consisted of mean measures, standard deviations, 95% confidence intervals, β values, *p* values, odds ratio risks, correlation coefficients, *p* trend values, standard errors, and prevalence ratios. Risk of bias was assessed for each study by examining sequence generation and selective outcome reporting. Blinding and other sources of bias were not included due to the nature of the study, which focused on the impact of dietary factors on metabolic outcomes. The risk of bias assessment was conducted by two independent researchers.

## 3. Results

### 3.1. Screening and Review Process

The search retrieved 1068 articles from the three databases used. Covidence deleted 84 duplicates. During title and abstract screening, conducted by two independent researchers and a tiebreaker, if conflicts emerged, 819 studies were excluded; 162 publications underwent full-text review. Again, two independent researchers reviewed each publication, and a tiebreaker resolved any disputes. In this step, 77 studies did not meet the inclusion criteria (see reasons in Figure 1), and the remaining 85 studies were used in this systematic literature review. All 85 studies included in this systematic review were cataloged using a table documenting the study characteristics (author, year, type of study, the data type, dietary intake, outcome variables used, and conclusions) (supplemental Table S1). Bias assessment results are reflected in Table S2. Results are presented by age group to assist in the understanding of the difference between dietary factors and age/developmental stage, which leads to the repeated mentioning of sources that reported data on all age groups (as compared to studies with a narrowly focused population). Also, race and ethnicity were not considered for this summary of results since many studies did not provide information on these characteristics of the samples.

### 3.2. Measuring Dietary Intake and Eating Patterns

Only studies using measured dietary intake or diet patterns were included in this review. Notably, there is considerable heterogeneity between the types of measurements across the studies. The cross-sectional studies from larger projects such as the National Health and Nutrition Examination Survey (NHANES [21,22,23]) and Healthy Lifestyle in Europe by Nutrition and Adolescents (HELENA [24,25,26]) predominantly based dietary data on food frequency questionnaires, or usual food intake behavior questionnaires, were employed to establish diet quality. Studies with smaller sample sizes used data from food diaries or 24-h recalls. In addition, some studies assessed cultural differences surrounding food availability and the diet quality of specific regions. For instance, Ritter et al. examined data sets of Brazilian adolescents in school in the Study of Cardiovascular Risk in Adolescents cohort and used a Brazilian Diet Quality Index [27]. The results are presented by the following age groups: 5- to 11-year-olds, 12- to 14-year-olds, and 15- to 18-year-olds. Some studies only reported aggregated results covering two or more of these age groups, which are presented as separate categories.

### 3.3. Measurement of Outcome Variables

The outcome variables focused on CVD risk factors varied between research reports. Some used guidelines established in position papers, such as those by the American Heart Association (AHA) [5]. Depending on the research questions, researchers used all, select, or a combination of risk factors. Most of the studies included blood pressure. Studies with larger sample sizes included systolic, diastolic, mean arterial, and overall blood pressure [12,28]. Another common grouping of outcome variables was blood lipid values, including TG, TC, HDL, and LDL. Most of these papers described cardiometabolic risk as a whole [29,30,31,32].

### 3.4. Diet and Blood Pressure (Systolic and/or Diastolic)

As noted above, blood pressure was the most common outcome variable used in the papers of this review. Blood pressure measures were presented in multiple forms, including whole blood pressure, systolic only [33,34], calculated mean arterial pressure [22,24,35,36], and classifications of whether the values were hypertensive or non-hypertensive [12,21,37,38,39]. Blood pressure was either compared to percentile charts or the adult normal, pre-hypertensive, or hypertensive values.

#### 3.4.1. Elementary School Age (5–11 Years Old)

In a prospective cohort study, Voortman et al. observed the relationship between protein intake at 1 year of age and BP at 6 years of age among 2841 Dutch children. Protein intake was categorized into first, second, and third tertiles, with mean intakes of 34.5 g/day, 41.7 g/day, and 50.2 g/day, respectively. In the children, the mean SBP value was 102 mmHg, while the mean DBP was 60 mmHg. In covariate-adjusted models, a higher protein intake (third tertile) was associated with lower DBP (0.09 SD (95% CI: −0.18 to 0.00); *p*-value = 0.4) compared with those in the first and second tertiles. However, no significant association with SBP was observed in the entire population. Sex-stratified analyses revealed a slightly stronger association among boys (*n* = 1384), where those in the third tertile had a 0.12 SD (95% CI: −0.23 to 0.00) lower DBP than those in the first and second tertiles. No significant associations were observed for SBP, nor among girls for DBP and SBP [40]. Among 1666 Dutch children aged five to six years old in a cohort study, Jaspers Faijer-Weste et al. evaluated ideal cardiovascular health (ICH) utilizing an extended ICH score that incorporated four ideal health behaviors (diet, physical activity, weight status, and smoking), three ideal health factors (glucose, total cholesterol, and blood pressure), and three extended health behaviors (sleep duration, screen time, and prenatal smoke exposure). The mean SBPs and DBPs in the extended ICH score were 105.2 mmHg and 60.1 mmHg, respectively. Thirty-three percent of the population scored an ICH score of 8–9, which was significantly associated with lower SBP (*p*-value = 0.012) and lower DBP (*p*-value 0.011) [41]. In a prospective cohort study of 3991 Dutch children aged eight years, Siddiqui et al. assessed dietary intake using a food frequency questionnaire. A diet quality score from 0 to 10 points was calculated based on adherence to age-specific nutritional guidelines. The children’s mean SBP and DBP values were 102.4 mmHg (95% CI, 97.2–107.7) and 58.0 mmHg (95% CI, 54.0–62.3), respectively. In models accounting for lifestyle and socioeconomic factors, as well as body mass index (BMI), higher diet quality was significantly associated with lower SBP with a 0.04 SD (95% CI: −0.06 to −0.01) and DBP with a 0.05 SD (95% CI: −0.07 to −0.02) [42]. A cross-sectional study conducted by Al-Farhan et al. of (*n* = 313) Kuwaiti fifth-grade children with a mean age of 10.4 ± 0.4 years had the main objective of determining whether poor healthy eating index (HEI) scores were associated with CVD risk, including elevated SBP or DBP and HTN. Elevated BP, defined as SBP or DBP ≥ 90th to <95th percentile or 120/80 mmHg to <95th percentile (whichever was lower), showed a weak positive association with lower HEI scores (HEI-2010: *r* = 0.121; *p* value = 0.35; HEI-2015: *r* = 0.146; *p* value = 0.011). However, no significant associations were reported between HEI scores and HTN [43].

In a longitudinal study of 2045 children in the Netherlands, Leermakers et al. examined early dietary exposures in relation to later cardiometabolic outcomes, reporting that a higher sugar-containing beverage intake at 13 months of age was associated with a higher cardiometabolic risk factor score at 6 years of age, with an SD of 0.13 (95% CI: 0.01–0.25). However, no significant associations were observed between each risk factor that comprised the overall cardiometabolic risk factor score, including BP, after adjustment in the total population or accounting for gender. Among boys, the direction of association for SBP or DBP trended in the direction of the higher cardiometabolic risk factor score but did not reach statistical significance [13]. In the Healthy Start Study, Perng et al. assessed overall cardiovascular health rather than the relationship between diet quality and BP among (*n* = 350) children aged four to seven years old; therefore, no direct associations between these metrics were reported [44].

#### 3.4.2. Middle School Age (12–14 Years Old)

Few studies were conducted in the 12–14-year-old age range specifically; however, all four studies summarized here measured BP and diet quality. Bodega et al. found no significant association between diet quality and BP in (*n* = 1326) Spanish adolescents [45]. Mustafa et al. assessed if breakfast frequency correlated to BP in (*n* = 795) Malaysian adolescents and found no significant association [11]. Okuda et al. in 2020 [31] conducted a study measuring the relationship between sugar intake and CVD risk factors in (*n* = 3242) Japanese adolescents. Increased sugar intake was found to be associated with increased SBP (P*_trend_* ≤ 0.025,1.7–2.3 mmHg), while no association was found with DBP [31]. Another study by Okuda et al. in 2021 [46] measured CVD risk factors in (*n* = 3162) Japanese adolescents. The sample was categorized based on the intake of the Japanese Food Guide Spinning Top (JFGST), which was created to promote healthy eating in Japanese adolescents. Data from this study showed that the highest adherence to the JFGST was associated with lower SBP compared to children with little or no adherence (*p* = 0.001). There was no significant difference in DBP between groups [46].

#### 3.4.3. High School Age (15–18 Years Old)

Three cross-sectional studies focusing solely on 15- to 18-year-olds include data on blood pressure. Burrows et al. and Murni et al. collected information on Chilean and Indonesian adolescents, respectively, but did not find any relationship between diet and BP. Burrows et al. found that the average BP was 112.2/69.3 (*n* = 667), with male participants having statistically higher mean values than female participants [47]. Murni et al. specified elevated BP as ≥120 mmHg in the obese Indonesian teenage population (*n* = 179). The median blood pressure was 115/73, which did not correlate with dietary quality and diet patterns. Seventy-nine percent of healthy Chilean adolescents had at least one CVD risk factor [10]. Agostinis-Sobrinho et al. measured the impact of the SEADiet, which includes a high consumption of fish, red meat, dairy products, vegetables and legumes, vegetable soup, potatoes, whole wheat bread, and wine. In the study of Portuguese adolescents (*n* = 467), mean systolic BP was 115.45 (±32.36). After controlling for confounding variables, the SEADiet was inversely correlated with SBP, TC, TG, and HDL [48]. Yap et al. studied overweight adolescents to examine diet and blood pressure (*n* = 108) and found a mean BP of 124/65 mmHg among the male participants. The range for SBP was 88–154, and DBP 45–89. Over half of the sample (58.3%) had elevated BP (SBP > 120 mmHg, DBP < 80) and 37.9% of participants had HTN (SBP ≥ 130 mmHg, DBP ≥ 80). Adjusted models indicated that each additional serving of fruit consumed was associated with 2.4 mmHg lower SBP (95% CI −4, −0.7) and 2.5 mmHg lower DBP (95% CI −3.9, −1.1). Vegetable intake was associated with lower DBP by 1.4 mmHg (95% CI −1.8, −0.4), and milk with 2.2 mmHg (95% CI −3.6, −0.8) lower DBP. Overall, there was no significant relationship between dietary intakes or habits and adjusted odds of HTN [49].

#### 3.4.4. Elementary, Middle School Age (5–14 Years Old)

Hur et al. used data from the Korean Child Adolescent Cohort study following children (*n* = 770). An average SBP of 97.3 mmHg (±10.2), DBP at 66.9 mmHg (±9.2), and a mean arterial pressure of 77 mmHg (±8.6) were reported. They found that there was not a direct relationship between diet and BP, as they were calculated as part of the continuous metabolic score [35]. Bull et al. analyzed data from the ALSPAC study (*n* = 2311) and examined different dietary patterns at lunch (healthy, processed, traditional, or packed lunch) with measured blood pressure values. In the unadjusted model, significant odds ratios for high BP risk, defined as being in the 90th percentile (SBP ≥ 133 mmHg), were found for the packed lunch at age 7 (OR = 1.49, *p* = 0.01), traditional diet at age 10 (OR = 1.38, *p* = 0.03), and packed lunch at age 13 (OR = 1.32, *p* = 0.0001). However, after adjustment, none of the associations were statistically significant. The risk of being in the 90th percentile for DBP (≥72 mmHg) followed a similar pattern in the unadjusted model for packed lunch at age 7 (OR = 1.44, *p* = 0.03) and packed lunch at age 13 (OR = 1.56, *p* = 0.01). Statistically significant patterns for processed lunch at age 7 (OR = 1.51, *p* = 0.01) and 13 (OR = 1.40, *p* = 0.02) were identified; however, after adjustment, the results were no longer significant [50]. Krijger et al. investigated the effect of DASH diet scores on BP patterns (*n* = 869). The results showed that adjusted higher DASH scores were associated with lower SBP (*p* = 0.046) and DBP (*p* < 0.001). In addition, logistic regression analysis between DASH scores at age five to six years old and risk of preHTN showed that a one-quintile increase in DASH score was associated with a lower risk of preHTN (aOR = 0.77, *p* = 0.012) [51]. Piernas et al. found that the prevalence of preHTN and HTN was approximately 20% and 14%, respectively, in Chinese school-age children (*n* = 663). Higher intakes of total daily calories (OR = 1.16, 95% CI 1.03, 3.18), total sugars (OR = 3.59, 95% CI 1.80, 6.14), and added sugars (OR = 2.13, 95% CI 1.22,3.74) were associated with an increased likelihood of preHTN. Furthermore, total (OR = 2.02, 95% CI 1.76, 7.32) and added sugars (OR = 2.64, 95% 1.11, 4.09) were associated with higher odds of HTN [52].

Shang et al. used machine learning to identify changes in diet that were associated with risk factors for cardiometabolic disorders (*n* = 5676) and found that healthy diet scores had inverse associations with DBP (*p* = 0.013). In all models, there were significant changes in risk factors attributable to baseline healthy diet scores. All adjusted models showed that, as the diet scores increased (higher scores corresponded to healthier scores), both SBP and DBP decreased (*p* < 0.0001). The healthiest diet scores were associated with lower SBP and DBP values (β of –0.46 (95% CI –0.58, −0.35) and β of –0.46 (95% CI −0.58, −0.34)), respectively [53].

A second study by Shang et al. examined how the clustering of low diet quality, low physical fitness, and unhealthy sleep patterns affected the cardiometabolic risk factor (*n* = 5315). There was no effect of diet quality on the combined outcome; however, low diet quality was positively associated with an adjusted cardiometabolic risk score difference (OR = 0.43 SD ± 0.14). The difference in cardiometabolic risk factors of children in the low versus high diet quality was 0.63 SD [54].

#### 3.4.5. Middle, High School Age (12–18 Years Old)

Yang et al. analyzed a cross-sectional study among (*n* = 18,757) Chinese adolescents aged 13 to 17 years to examine the relationship between vegetable consumption and BP. The reported daily vegetable intake among the subjects varied, with 12.2% consuming less than one serving, 38.0% consuming one to two servings, 28.7% consuming two to three servings, and 21.1% consuming three or more servings per day. A significantly lower likelihood of high BP was correlated with consuming at least three servings of vegetables daily compared to those consuming less than one serving per day (OR = 0.74; 95% CI: 0.58 to 0.94; *p* = 0.013) [55]. In Sethna et al.’s cross-sectional analysis among a nationally representative sample of U.S. adolescents aged 12–18 years from the National Health and Nutrition Examination Survey (NHANES) cycles from 2005 to 2014, the association between the Children’s Dietary Inflammatory Index (C-DII) and BP was examined. The average C-DII score was 0.86 (SE 0.04), indicating a slightly pro-inflammatory dietary pattern. In adolescents with obesity, increasing C-DII quartiles were associated with significantly higher SBP (β = 5.07; 95% CI: 2.55 to 7.59), while a pro-inflammatory diet was associated with lower DBP (β = −4.14; 95% CI: −6.74 to −1.54) [23].

An observational study conducted in India by Pusdekar et al. examined (*n* = 200) adolescents aged 13–17 years to assess the relationship between dietary salt consumption and BP. Among the cohort, the mean SBP was 113 ± 16 mmHg and mean DBP was 73 ± 12 mmHg. Individuals who consumed high levels of salt had a 1.72-fold higher risk (95% CI: 1.5 to 1.9) of pre-HTN or HTN, and those who frequently consumed “junk food” had a 1.4-fold increased risk (95% CI: 1.23 to 1.56) [56]. Neves et al. investigated the association between soft drink consumption and CVD risk in a cross-sectional study of (*n* = 36,956) Brazilian adolescents aged 12 to 17 years. After adjustment for covariates, consuming ≥450 mL of soft drinks per day was significantly associated with HTN (OR = 1.22 to 1.26; 95% CI: 1.01 to 1.53; *p* value = 0.02 to 0.04) [39].

In a cross-sectional study involving (*n* = 548) European adolescents, Perez-Gimeno et al. examined the interaction between the Mediterranean Diet (MedDiet) score and BP as well as whether this relationship was modified by genetic susceptibility through an HTN–genetic risk score (HTN-GRS), which was based on multiple BP-related single-nucleotide polymorphisms, and its association with HTN. The mean SBPs and DBPs in the cohort were 117 mmHg (range = 108–124 mmHg) and 64 mmHg (range = 59–70 mmHg), respectively, which corresponded to z-scores of 0.65 (range = 0.02 to 1.30) for SBP and 0.63 (range = −0.10 to −1.20) for DBP. A significant interaction was found between HTN-GRS and MedDiet adherence for both SBP (β = 0.02; *p* < 0.001) and DBP (β = 0.02; *p* < 0.001). Moreover, adherence to the MedDiet was associated with lower z-SBP (β = −0.40; *p* < 0.001) and z- DBP (β = −0.29; *p* = 0.001) [25]. In a longitudinal study with Australian participants aged 14 to 17 years old, Appannah et al. evaluated the associations between an “energy-dense, high-fat, and low-fiber” dietary pattern and cardiometabolic risk factors during adolescence, including the tracking of DBP during adulthood. Dietary intake, anthropometric measures, and biochemical markers were collected, dietary pattern z-scores were derived from reduced rank regression, and the tracking of DBP z-scores was assessed utilizing Pearson’s correlation coefficient. The DP demonstrated moderate tracking between ages 14 and 17 (r = 0.51 and 0.45 for boys and girls, respectively) [57]. Madalosso et al. examined the association between UPF consumption and cardiometabolic risk factors, including BP, in a cross-sectional study sample of (*n* = 36,952) adolescents aged 12–17 years who participated in the Study of Cardiovascular Risk in Adolescents (ERICA) study. In partially adjusted models, UPF consumption showed an inverse association with high BP (PR = 0.878; 95% CI: 0.801–0.963), indicating that among individuals with higher UPF intake, there is a lower prevalence of elevated BP, although this result is statisically insignificant in other models of adjustment [58]. Agostinis-Sobrinho et al. found no significant association between diet quality and SBP. Diet quality was measured as adherence to the Mediterranean diet [59]. Moreover, an examination study by Hecht et al. assessed trends in overall CVD indicators, such as BP, among U.S. adolescents aged 12 to 16 years old; yet, they did not focus on the specific biomarker relationship between diet and BP [60].

#### 3.4.6. Elementary, Middle, High School Age (5–18 Years Old)

Many studies only reported results that integrated all age groups. Buckland et al. measured dietary data in a complete case analysis in (*n* = 2270) children aged 7, 10, and 13, and then measured similar data as well as identified a CMR score in (*n* = 1058) adolescents aged 17 and (*n* = 1070) adults aged 24. They found a decrease in the odds of high DBP at age 17 (OR = 0.89, 95% CI 0.80, 0.98) for each increase in adherence to the Mediterranean diet at age 7. Researchers found a significant association between a high Mediterranean diet score and CMR risk from ages 13 to 24, with a 32% (OR 0.68 (95% CI: 0.49, 0.94)) decrease in odds of having a high CMR score. Although MAP (mean arterial pressure) was measured, other criteria, such as DBP, had a more significant influence [61]. Macknin et al. conducted a 4-week prospective study (*n* = 30) that randomized groups of children to plant-based (PB) and American Heart Association (AHA) diets. Children in the PB group had a significant (*p* < 0.05) reduction in SBP from baseline (−6.43 mmHg) [33]. Gilardini et al. found in (*n* = 448) obese children that, after adjusting for waist/height, both DBP and SBP were inversely associated with vegetable protein intake (systolic r = −0.120; *p* < 0.05; diastolic r = −0.267; *p* < 0.01) [62]. A study on daily salt consumption by Emamian et al. in (*n* = 1455) Iranian children found a positive association between salt intake and SBP, with a linear relationship of 0.41 (95% CI 0.17, 0.65) [63]. Latorre-Millan et al. conducted a study measuring the health markers of (*n* = 674) participants who fell under the categories of health-conscious (HC) and sweet and processed (SP) food patterns and found a significant difference in mean DBP between HC and SP in the total sample, correlating lower DBP to better diet quality (*p* ≤ 0.05) [64]. Fulgoni et al. measured the association between dietary fiber and risk factors for CVD using data from the NHANES study. They found that the risk of elevated DBP decreased by 23% (OR 0.77, P_linear_ trend = 0.0210) with increased fiber density and also a decrease in DBP with an increased absolute fiber intake of 10% (OR 0.90, P_linear_ trend = 0.0307) [22]. In a study by Mohan et al. conducted on (*n* = 1959) North Indian adolescents, it was found that added salt intake had a significant association with risk of HTN, defined as BP > 95 percentile (RRR 4.90, 95% CI 2.83, 8.48) [12]. Ramadas et al. compared dietary intake and CVD risk factors in a sample of (*n* = 623) Southeast Asian children and adolescents. In children with adherence to fish and seafood dietary recommendations, a decrease in median SBP (107 mmHg vs. 110 mmHg, *p* = 0.001) [65] was reported. Wang et al. in 2022 conducted a study in China on (*n* = 10536) individuals aged 7–18, measuring the effect of soy intake on HTN and obesity, and found that high soy food intake and high soy food frequency was associated with reduced SBP (*p* < 0.001), and there was no association found with DBP [66]. Yang et al. in 2024 [38] measured BP in (*n* = 3150) children and adolescents who ate fresh fruit and vegetables every day and found that they had the lowest prevalence of HTN (*p* < 0.05). Skipping breakfast was associated with a higher prevalence of HTN (*p* < 0.01) [38]. Several studies found no significant results: Aljahdali et al. found no significant difference between SBP and DBP in (*n* = 574) participants aged 8–21 years old following the DASH or Mediterranean diets [67]. Aparicio-Cercós et al. [68] investigated the effects of the Mediterranean diet on a sample of (*n* = 4402) adolescents, Banerjee et al. [69] measured BP related to the consumption of UPF and vegetable/fruit intake in a sample of (*n* = 814) adolescents, Velazquez-Lopez et al. [29] conducted a study measuring the effects of the Mediterranean diet on a sample of (*n* = 49) obese children and adolescents, and Çağiran Yilmaz et al. [70] measured the effects of the Mediterranean diet on BP with a sample of (*n* = 95) adolescents in Turkey. Dong et al. measured the association between snacking fruits and vegetables compared to other snacks, with their final analytical sample size being (*n* = 3875) children [71]. Hu et al. found no significant relationship between diet quality and MAP in a sample of (*n* = 192 adolescents) [36]. Toft et al. found no significant difference in SBP or DBP in (*n* = 156 children) with a reduced salt intake [72]. Seral-Cortes et al. compared obesity-related genetic risk scores (GRSs) and Mediterranean diet (MD), finding that MD in males had a significant effect on DBP (*p* < 0.05) and that, in females, it had a significant effect on SBP (*p* < 0.05). In males, there was also a significant association between MD and MAP (*p* < 0.05) [26]. Menghetti et al. conducted a study on Italian school children and found that an unhealthy diet was associated with increased rates of HTN (OR: 1.43; 95% CI: 0.89–2.29) [37].

### 3.5. Diet and Lipoproteins (HDL, LDL, TC)

#### 3.5.1. Elementary School Age (5–11 Years Old)

Rauber et al. conducted a longitudinal cohort study among (*n* = 305) Brazilian children aged 7 to 8 years old from low socioeconomic backgrounds to assess the effects of the early consumption of processed and ultra-processed foods (UPFs) on lipid profiles from preschool to school age. At the follow-up age of 7 to 8, the mean TG, LDL, and HDL concentrations were 161.9 mg/dL, 100.2 mg/dL, and 48.0 mg/dL, respectively. After adjustment for confounders, preschool-age consumption of UPF was significantly associated with increases in both TG (β = 0.430 mg/dL per 1% increase in energy intake; 95% CI: 0.008–0.853; *p* value = 0.046) and LDL (β = 0.369 mg/dL per 1% increase in energy intake; 95% CI: 0.005–0.733; *p* value = 0.047) at school age [73].

A population-based cohort study performed in the Netherlands by van Gijssel et al. examined associations between dietary fiber intake during infancy and cardiometabolic health in childhood among (*n* = 2032) children. After adjustment for parental and child characteristics, a higher energy-adjusted nutritional fiber intake by 1 g/day was associated with a 0.026 standard deviation score (SDS) increase in HDL (95% CI: 0.009–0.042), correlating to an approximate 0.31 mg/dL increase. Additionally, a 1 g/day increase in fiber intake from fruits and vegetables was significantly associated with a 0.028 SDS increase in HDL (95% CI: 0.001–0.054). No significant associations were found between dietary fiber intake and TG or LDL [74]. Giannini et al. conducted a 12-month intervention study to examine the relationship between the influence of the Mediterranean diet and the variation in lipoprotein values in (*n* = 35) pre-pubertal children with hypercholesterolemia. At baseline, the children had a mean TC level of 260.6 ± 51.2 mg/dL, HDL of 53.0 ± 15.6 mg/dL, and LDL of 182.0 ± 53.8 mg/dL. After 12 months of Mediterranean diet intervention, a significant reduction in TC and LDL cholesterol was observed (both *p* < 0.001), as well as a substantial increase in HDL cholesterol (*p* < 0.05). Moreover, at six months of follow-up, LDL was still significantly lower (*p* < 0.05) [75].

In a cross-sectional study of (*n* = 1948) schoolchildren 9 to 10 years old in the United Kingdom, Donin et al. investigated the associations between the frequent consumption of takeaway (take-out) meals and coronary heart disease risk markers, including adverse lipid profiles. A significant association was observed between takeaway meal consumption and both TG (*p* = 0.04) and LDL (*p* = 0.01). Furthermore, children who consumed a takeaway meal at least once per week had TG levels that were 0.09 mmol/L higher (95% CI: 0.01–0.18) and LDL levels that were 0.10 mmol/L higher (95% CI: 0.02–0.18) compared to children who rarely or never consumed takeaway meals. Moreover, the associations between takeaway meal consumption and total and LDL cholesterol levels remained statistically significant after adjustment for fat mass index and did not differ by ethnicity [76]. After assessing diet quality among (*n* = 204) boys and (*n* = 198) Finnish girls aged 6–8 years old and calculating DASH, Baltic Sea Diet (BSDS), Mediterranean Diet, and Finnish Children Healthy Eating Index (FCHEI) scores, Eloranta et al. calculated the associations between diet and CVD risk. Among girls, greater adherence to healthier dietary patterns, reflected by higher DASH and BSDS scores, was associated with lower HDL levels (DASH: β = −0.19; *p* value = 0.011; BSDS: β = −0.23; *p* value = 0.001). No significant associations were observed for LDL or in boys [77]. Ahola-Olli et al. investigated whether genetic variation modified the association between dietary fat intake and serum lipid profiles among (*n* = 483) Finnish children aged 5 to 7 years. While this study supported the connection between dietary fat and LDL, the primary emphasis was on gene–diet interactions. Yet, at age 5, mean LDL levels were 2.84 ± 0.65 for boys and 2.87 ± 0.65 for girls (*p* value = 0.710), HDL levels were 1.37 ± 0.27 for boys and 1.39 ± 0.26 for girls (*p* value = 0.423), and TG levels were 0.76 ± 0.27 for boys and 0.77 ± 0.27 for girls (*p* value = 0.733). Moreover, at age 7, LDL levels were 2.86 ± 0.65 for boys and 2.88 ± 0.63 for girls (*p* value = 0.756), HDL levels were 1.34 ± 0.26 for boys and 1.34 ± 0.25 for girls (*p* value = 0.925), and TG concentrations were 0.77 ± 0.28 for boys and 0.84 ± 0.32 for girls (*p* value = 0.033) [78].

#### 3.5.2. Middle School Age (12–14 Years Old)

A study by Bodega et al. measured the relationship between diet quality and CVD risk factors in (*n* = 1326) middle-school-aged children. Children in the “healthy” diet category were found to have significantly higher TC (mean 157.6 mg/dL, 95% CI: 150.7, 164.4) compared to children in the “processed” diet category (mean 148.6 mg/dL, 95% CI:141.6, 155.5); this trend was attributed to the differences in non-HDL cholesterol levels, with “processed” having significantly lowered non-HDL compared to “healthy” diets (*p* = 0.027) [45]. Mustafa et al. conducted a study comparing breakfast frequency to CVD risk factors in (*n* = 795) children. After adjusting for potential confounders, each extra day of breakfast was associated with a lowered TC concentration of −0.03 mmol/L (95%CI 0.06, 0.01), attributed to lowered LDL levels of (β −0.03, 95%CI 0.06, 0.01) [11]. Okuda et al. in 2020 compared sugar intake and CVD risk factors in (*n* = 3242) children and showed no significant association between sugar intake and cholesterol levels [31]. Okuda et al. in 2021 conducted a study on (*n* = 3162) Japanese adolescents measuring adherence to Japanese Food Guide Spinning Top (JFGST) recommendations and found no significant changes in HDL or LDL levels based on adherence to the JFGST guidelines [46].

#### 3.5.3. High School Age (12–14 Years Old)

Murni et al. measured a median LDL of 117 mg/dL and found that there was no relationship with diet quality. The total cholesterol in the population of obese Indonesian adolescents (*n* = 179) had a median of 174 mg/dL and had no relationship with any diet aspects. The median HDL for this population was 44 mg/dL, and there was a statistically significant correlation between fiber intake and HDL level (β = 0.165, *p* = 0.033) [10]. Agostinis-Sobrinho et al. reported a study looking at the Southern European Atlantic diet and reported a mean TC in the population of 162.38 (±10.40) (*n* = 467); females had higher TC than male participants (*p* < 0.01) and girls presented with higher HDL (*p* < 0.001) than boys (mean HDL was 55.62 mg/dL (±13.1)). There was no association with diet [48]. Burrows et al. also found no relationships of lipoproteins with dietary factors, but 69.9% (95% CI: 66.4, 73.4) of the study population had low HDL and females had a higher prevalence of low HDL (*p* < 0.01) than male participants (*n* = 667) [47].

#### 3.5.4. Elementary, Middle School Age (5–14 Years Old)

Hur et al. grouped information on HDL and TC into the relative weighted score of the continuous metabolic syndrome score and reported a mean TC of 169.8 mg/dL (±26.4) and HDL at 58.8 mg/dL (±11.2) *(n* = 770). Data showed that sugar consumption from SSBs increased metabolic syndrome score (β = 0.04, *p* = 0.02), but this disappeared after follow-up [35]. Bull et al. calculated the odds of being in the 90th percentile for LDL (≥0.59 mmol/L), HDL (≤0.92 mmol/L), and TC (≥4.67 mmol/L) (*n* = 2311). For LDL, there were no significant patterns in the unadjusted model but the adjusted model indicated a significantly lower risk when following the healthy dietary pattern at age 7 (OR = 0.54, 95% CI 0.32, 0.90) and at age 10 (OR = 0.53, 95% CI 0.32, 0.87). HDL had a significant increase in the unadjusted risk analysis following the traditional diet pattern at age 7 (OR = 1.54, 95% CI 1.07, 2.21) and the relationship remained after adjustment (OR = 1.83, 95% CI 1.09, 3.05). The unadjusted model at age 13 for both healthy (OR = 1.4, 95% CI 1.01, 1.94) and processed diets (OR = 1.5, 95% CI 1.04, 2.15) had an increased risk for low HDL values, but these trends disappeared after adjustment. The unadjusted model for TC at age 13 showed a decrease in the risk of high TC for the traditional diet (OR = 0.65, 95% CI 0.44, 0.98); however, this trend was not present after adjustment. In addition, the adjusted model showed a significant decrease in the risk of high TC for the processed (OR = 0.50, 95% CI 0.3, 0.84) and the traditional diet (OR = 0.41, 95% CI 0.23, 0.73) [50]. Costa-Urrutia et al. conducted an intervention that included physical activity, health education, parent involvement, and school meals in urban and indigenous schools (*n* = 320) and reported a decrease in TC from baseline to follow-up in girls (β = −16.86, *p* = 0.00026) and in one of the indigenous schools (β = −9.99, *p* = 0.02). LDL levels in girls decreased from baseline to follow-up (β = −4.09, *p* = 0.05), and those in the overweight–obesity group had an increase in LDL (β = 5.27, *p* = 0.02). HDL decreased after treatment (β = −4.03, *p* = 0.01) [79]. Dennison et al. examined the prevalence of risk in the American Indian population for obesogenic behaviors and the consumption of fruit and vegetable intake and sugar-sweetened beverages. (*n* = 121) and found a 57% risk for low HDL and 8% risk for high LDL [30]. Krijer et al. found no significant associations between diet quality and LDL, HDL, and TC (*n* = 869) [51]. Lahoz-García explored the effect of dairy intake and found that children who had normal values for HDL consumed more whole milk compared to those with low (<40 mg/dL) HDL (*p* = 0.004) in a minimally adjusted model and in the further adjusted model (*p* = 0.011) (*n* = 1088). Those with normal HDL consumed less semi-skimmed milk in a minimally adjusted model (*p* = 0.001) and a further adjusted model (*p* = 0.002). No differences were found with total cholesterol or LDL [80]. Martino et al. examined the relationship between the Mediterranean score (Kid-Med) and TC (*n* = 29,159). The Kid-Med score was significantly and inversely related to TC (β = −0.066, *p* = 0.032) [81]. Piernas et al. found that 21% of children in the study population (*n* = 663) had dyslipidemia (≥1 lipid measurement exceeding high lipid cut points) and that more than half of the children had pre-dyslipidemia (defined at ≥1 lipid measurement above borderline levels). Prevalence estimates were 23% for TC, 13% for LDL, and 14% for HDL. There were no significant correlations between LDL, HDL, and TC with any level of dietary intake in all categories [52]. Shang et al. found, in a study of children (*n* = 5676) investigating the effect of diet quality on CMR risk, an increase in both LDL (*p* < 0.0001) and HDL (*p* = 0.0012) at baseline. At follow-up, HDL had increased in individuals who had a higher diet score at baseline (*p* < 0.0001) in all models [53].

#### 3.5.5. Middle, High School Age (12–18 Years Old)

A cross-sectional study conducted by Ochoa-Avilés et al. evaluated the association between dietary patterns and their relationship with CVD risk among (*n* = 779) adolescents in grades 8–10 from both urban and rural Ecuador from different socioeconomic backgrounds. Adherence to a wheat-dense, animal-fat dietary pattern, characterized by a high intake of refined wheat products, red meat, animal fat, dairy, and plantains, along with a low consumption of maize and whole grains, was significantly associated with higher TC (*p* value = 0.02) and LDL (*p* value = 0.04) among rural participants [28]. Agostinis-Sobrinho et al. conducted a study (*n* = 2477) to determine if diet quality can overcome the effect of a lack of fitness when assessing CVD risk and found that high adherence to the Mediterranean diet (compared to low adherence) significantly increased HDL levels (*p* < 0.05), though they found no significant relationship with LDL [59]. A randomized placebo-controlled clinical trial by Sarf-Bank et al. conducted at the Pediatric Cardiovascular Research Center in Isfahan, Iran, studied the relationship between curcumin supplementation and CVD risk markers in (*n* = 60) overweight and obese adolescent girls aged 13 to 18 years who were randomly assigned to receive either curcumin or a placebo for 10 weeks. Curcumin supplementation was associated with an increase in HDL (*p* value = 0.042); however, the univariate analysis of covariance (ANCOVA) showed no significant differences between the intervention and placebo groups after 10 weeks of supplementation (*p* value > 0.05) [32]. Ritter et al. examined the association between diet quality, measured with the Diet Quality Index for Adolescents adapted for Brazilians (DQIA-BR), and cardiometabolic markers among (*n* = 36,959) Brazilian adolescents aged 12 to 17 years old enrolled in the Study of Cardiovascular Risks in Adolescents (ERICA) cross-sectional study. Higher DQIA-BR scores indicated better diet quality and were associated with lower LDL (β = −0.227; 95% CI: −0.448 to −0.005). Among boys with overweight or obesity, better diet quality was also associated with lower TC (β = −0.338; 95% CI: −0.611 to −0.066) [27]. Madalosso et al. utilized a cross-sectional study to examine the association between UPF consumption and cardiometabolic risk factors among (*n* = 36,952) Brazilian adolescents aged 12–17 who participated in the ERICA study. After adjusting for potential confounders, higher UPF consumption was associated with elevated LDL (PR = 1.012; 95% CI: 1.005 to 1.029) and inversely related to low HDL (PR = 0.972; 95% CI: 0.952 to 0.993) [58]. Using a cohort of (*n* = 236) European participants aged 12.5 to 17.5 years, Morcel et al. investigated the impact of nutritional and activity-related characteristics during adolescence on CVD risk in adulthood, where the participants were reassessed as young adults between 21 and 32 years of age. A higher intake of UPF foods during adolescence was associated with lower non-HDL cholesterol (*p* value = 0.003), while higher Diet Quality Index and Planetary Health Diet Index scores were associated with higher HDL in young adulthood (*p* value = 0.014; *p* value = 0.016, respectively) [82]. Saber et al. found that the Mediterranean diet score (MDS) during adolescence impacted cardiometabolic outcomes in adulthood among (*n* = 668) participants aged 10–19 years from the Tehran, Iran Lipid and Glucose Study. Using a validated food frequency questionnaire, the MDS was calculated based on eight dietary components. Over a 6.8-year follow-up, a higher MDS was inversely associated with changes in HDL and TC (*p* trend < 0.05). After adjusting for potential confounders, individuals in the highest tertile of MDS had a significantly lower risk of high TC (HR = 0.36; 95% CI: 0.18 to 0.74; *p* value = 0.004) compared to those in the lowest tertile [15].

#### 3.5.6. Elementary, Middle, High School Age (5–18 Years Old)

A longitudinal analysis conducted by Aljahdali et al. on (*n* = 574) Mexican children and adolescents found a positive correlation between HDL and adherence to a Mediterranean diet in boys. A significant increase in serum HDL values (*p* < 0.00625) was found in boys with the highest adherence to the Mediterranean diet [67]. A study by He et al. found a positive association between fish intake and HDL (0.06 SD (95% CI: 0.01 to 0.10)) [83]. Latorre-Millan et al. found in normal-weight children (BMI 17.3 ± 2.3) a significant difference in the mean HDL/LDL ratio between children falling under the “health conscious” vs “sweet and processed” diets (*p* ≤ 0.01) [64]. Lehtovirta et al. measured diet and lipid levels from infancy to age 7 biannually; then, from age 7 to 20, data was measured annually. Diet quality was measured by a point system where points were given for subjects with a low saturated-to-unsaturated-fat ratio, energy from saturated fat below 10%, dietary fiber ≥80th age-specific percentile, and sucrose ≤ 20th age-specific percentile. Participants with at least one target point had lower concentrations of TC and LDL (*p* = 0.004) as well as a decrease in LDL particle size (*p* = 0.003) [84]. The study conducted by Macknin et al. measuring lipid profiles during plant-based (PB) and AHA diets found significant changes from baseline in TC, LDL, and HDL. During PB, LDL and TC changed (−13.14 mg/dL and −22.5 mg/dL, respectively). In the AHA diet, HDL decreased (−2.93 mg/dL) [33].

Ramadas et al., in a study on Southeast Asian children, found that children with low HDL levels consumed more meat, poultry, and eggs (1.7 servings/day vs. 4.0 servings/day, *p* = 0.011) [65]. Beck et al. found no significant correlation between adherence to the DASH diet and lipoprotein levels; however, there was a positive association between an increase in protein and LDL values [85]. Çağiran-Yilmaz et al. found no association between diet (based on the KIDMED scale) and HDL/LDL levels [70]. Fulgoni et al. found no association between dietary fiber or whole grain intake and HDL or LDL levels [22]. Velazquez-Lopez et al. conducted a study measuring MD vs. standard diet. MD was significantly associated with an increase in HDL (*p* =0.001), decrease in TC (*p* = 0.001), and decrease in LDL (*p* = 0.001), with participants consuming the standard diet having no significant difference [29]. Winpenny et al. conducted a longitudinal study measuring age-related metabolic risk and diet quality. In adolescents, there was a significant association between HDL levels and fruit and vegetable biomarker score (*p* < 0.05) [86].

### 3.6. Diet and TGs

#### 3.6.1. Elementary School Age (5–11 Years Old)

Voortman et al.’s prospective cohort study in the Netherlands investigated whether protein intake at one year was associated with changes in TGs at six years of age among 2841 children. Children were grouped into tertiles based on daily protein consumption, with mean intakes of 34.5 g/day, 41.7 g/day, and 50.2 g/day, while TG levels ranged from 0.40 to 2.36 mmol/L. After adjusting for relevant child and parental factors, a higher protein intake was associated with lower TGs in the overall sample, with a 0.07 SDS decrease (95% CI: −0.13 to −0.01), with the most substantial effect observed in the highest protein tertile group, with a 0.14 SDS decrease (95% CI: −0.24 to −0.03; *p* value = 0.01). Moreover, each additional 10 g/day of protein corresponded to a 0.12 SD decrease in TGs (95% CI: −0.20 to −0.04) among boys, where the association between protein intake and TGs was primarily observed [40]. In a population-based cohort of 2032 Dutch children, van Gijssel et al. investigated whether dietary fiber intake during infancy was associated with cardiometabolic outcomes later in childhood. After adjustment for relevant parental and child factors, each 1 g/day increase in energy-adjusted dietary fiber intake corresponded to a 0.020 SDS lower TG level (95% CI: −0.037 to −0.003), equating to an approximate 0.89 mg/dL decrease in TGs per 1 g/day of increase in fiber intake [74].

Eloranta et al. assessed diet quality in (*n* = 204) boys and (*n* = 198) girls aged 6 to 8 years in Finland. They applied the DASH, Baltic Sea diet, and Mediterranean diet scoring systems, along with the Finnish Children’s Healthy Eating Index (FCHEI), to quantify dietary patterns for analysis on the associations between diet quality and cardiometabolic risk factors, including TGs. In boys, higher adherence to healthier dietary patterns was reflected in higher DASH and FCHEI scores, which were associated with lower TG levels (DASH: β = −0.16, *p* = 0.023; FCHEI: β = −0.17, *p* = 0.014). Furthermore, among girls, higher FCHEI scores were associated with lower TG concentrations (β = −0.16; *p* value = 0.033), with no association observed between DASH scores and TG levels [77].

#### 3.6.2. Middle School Age (12–14 Years Old)

Mustafa et al. found no significant correlation between breakfast frequency and TG levels [11]. Another study by Okuda et al. measured sugar intake in middle-school-aged children as a percentage of energy intake (%E). Children in higher quintiles for %E had no significantly different TG levels than children with intakes in the lower quintiles [31].

#### 3.6.3. High School Age (15–18 Years Old)

Burrows et al. defined the CVD risk factor for hypertriacylglycerolaemia as ≥150 mg/dL. The mean TG value was 88.3 mg/dL for their participants (*n* = 667). There was no relationship with food quality intake reported [47]. Agostinis-Sobrinho et al. reported a mean value of 71.29 (±37.9) mg/dL but found no relationship directly with SEAdiet measurements (*n* = 467) [48]. Murni et al. reported a 114 mg/dL TGs median for their study population (*n* = 179). There was no reported relationship for usual diet and TGs [10].

#### 3.6.4. Elementary, Middle School Age (5–14 Years Old)

Hur et al. reported the mean TG of 63 mg/dL (42–92) for their study participants (*n* = 770) but did not find any relationship with diet when grouping TGs into a continuous metabolic syndrome score [35]. Bull et al. looked at the risk of being in the 90th percentile for TG (≥1.29 mmol/L) (*n* = 2311), and, after adjustment, the healthy diet pattern at age 7 (OR = 0.53, 95% CI 0.32,0.89) and age 10 (OR = 0.52, 95% CI 0.32, 0.87) was inversely associated with being in the 90th percentile. In addition, adjusted models showed a decreased odds ratio of being in the highest TG percentile and the processed food pattern at age 10 (OR = 0.58, 95% 0.35, 0.95). Neither of these patterns was seen in the unadjusted models [50]. Costa-Urrutia et al. demonstrated that, between baseline and post-intervention (physical activity, school meals, health education, and parental involvement intervention), a decrease in TGs was found, especially in the overweight–obesity category (β = −9.99, *p* = 0.03) (*n* = 320) [79]. Dennison et al. established in a sample of American Indian children that 17% had high TGs, but reported no link with adherence to healthy diet guidelines (*n* = 121) [30]. Krijger et al. demonstrated that TG levels decreased at ages 5 and 6, with a better DASH score (*p* = 0.032) and a better child diet quality score (*p* = 0.044) (*n* = 869). At follow-up, DASH and CDSQ scores at ages 5 to 6 were associated with the risk of CVD at ages 11–12. DASH scores and dyslipidemia had an adjusted OR of 0.79 (95% CI 0.65, 0.95), and CDQS and dyslipidemia had an OR of 0.79 (95% CI 0.66, 0.95) [51]. Lahoz-García et al. tracked different types of dairy intakes and the differences between those with normal and high TGs. In the minimally adjusted model, those with normal TGs consumed more whole-fat milk (*p* = 0.048) than those with high TGs; however, this trend was not preserved when further adjusted (*n* = 1088). Additionally, those with normal TG levels consumed less semi-skimmed milk (*p* = 0.006) in the minimally adjusted model. This trend held when the model was further adjusted for more confounders (*p* = 0.023) [80]. Piernas et al. established a prevalence of high TGs in their target population of 17% (*n* = 663). They found no statistical significance with TG levels and dietary intake pattern (low, medium, and high) [52]. The computer model established by Shang et al. did not find significant results from changes in healthy diet score and TG levels at baseline and follow-up (*n* = 5676) [53].

#### 3.6.5. Middle, High School Age (12–18 Years Old)

Agostinis-Sobrinho et al. measured whether diet could overcome the effects of poor fitness among 2477 adolescents aged 12–18 years old. This study compared CVD risk factors based on adherence to the Mediterranean diet (MeDiet). Children who reported high MeDiet scores had lower TG levels on average, with a mean TG of high MeDiet scores at 76.6 (±37.7) and low MeDiet scores at 87.9 m/dL (±42.1) [59].

#### 3.6.6. Elementary, Middle, High School Age (5–18 Years Old)

In a study conducted by He et al., who investigated CVD risk factors over time, it was found that fish consumption was associated with TG levels –0.07 SD (95% CI: −0.11 to –0.02) [83]. Hu et al. measured CVD risk factors over pubescent years, obtaining baseline measurements at age 12.9 ± 1.88 and follow up measurements at age 14.9 ± 1.91. When stratified for puberty development, there was still an inverse association between diet quality and TG z-score (β = −0.022, *p* = 0.01) [36]. Maffeis et al. found that diets with increased fat content higher than 35% of the total energy had a significantly higher chance [OR = 3.333 (95% CI: 1.113–9.979), *p* = 0.031] of TG/HDL > 2.2, with the significance increasing [OR = 4.804 (95% CI: 1.312–17.593), *p* = 0.018] if the saturated fat intake was greater than 13% of the total energy [87]. A study by Fulgoni et al. found that the risk of elevated TG decreased by 52% (OR = 0.48, Plinear = 0.0116) with increasing whole grain density [22]. Aljahdali et al. examined the effect of DASH, MedDiet, and C-DII index scores at three time points in the participant’s life. At each time point, SBP, DBP, TG, and HDL were measured. Researchers found that, for every unit increase in MedDiet score, there was a 3% reduction in serum TG. DASH and CDII scores were found to have no association with TG levels [67]. Velazquez-Lopez et al. investigated the effect of the Mediterranean diet on lipid levels in obese children and adolescents (*n* = 49). Data was collected at baseline and at week 16 of the intervention. The results showed a significant decrease in TG levels from baseline (*p* = 0.001) [29]. Ramadas et al. investigated the association of dietary intake using food frequency questionnaires for different food groups (cereals and cereal products; fruits; veggies; meat/eggs; seafood; legumes, nuts, and seeds; dairy; processed foods/ beverages) and measured CVD risks factors TC, TG, HDL, and LDL in (*n* = 162) Malaysian children and adolescents. Females with elevated TG reported consuming fewer fruits (0.8 servings/day vs. 1.2 servings/day, *p* = 0.011) but a higher intake of fish and seafood (2.3 servings/day vs. 0.9 servings/day, *p* = 0.042) compared to non-elevated TG participants [65]. Çağiran Yilmaz et al. found no association between TG levels and diet quality, although they did find BMI to be an influencing factor [70]. Lehtovirta et al. and Macknin et al. both found no significant association between diet quality and TG levels [33,84]. Another study that found no significant association between Mediterranean diet (MD) and TG levels was by Seral-Cortes et al., who studied the effects of MD and obesity genetic risk score on CVD risk factors [26].

Overall, 66% of studies measuring BP found an association between diet and either SBP or DBP among either male or female children aged 5–18; 50% of studies reported an association between diet and TG levels among either male or female children aged 5–18, and 74% of the studies found an association between diet and measured HDL, LDL, or TC among either male or female children aged 5–18.

## 4. Discussion

Cardiovascular disease affects many individuals of all ages and is the most common cause of death [1]. One of the most overlooked opportunities to improve public health by reducing the risk for CVD is a focus on the modifiable lifestyle risk factor of dietary intake to reduce the development and support treatment of CVD [2]. Since children also exhibit risk factors for CVD and dietary intake patterns track from childhood into adulthood, it is critical to better understand the relationship between diet and CVD risk factors in childhood. This systematic literature review was conducted to address this issue by summarizing current knowledge on the relationship between diet and CVD risk factors in children aged 2–18 years old published in English.

The results showed that a plethora of studies have been published on this topic, especially in children older than five years. However, most studies did not include measured or self-reported dietary intake data, used specific intake patterns or indicators of overall diet quality, or included measured blood levels of lipoproteins or blood pressure. Our summary includes 85 research papers that elucidate the highly complex relationship between dietary factors and CVD risk and demonstrate the need for more consistent study designs, especially definitions of dietary and outcome factors.

One challenge in summarizing across different studies is the large heterogeneity in dietary assessment tools employed to calculate intake data. Several publications are available that provide in-depth discussion of the strength and weaknesses of the intake assessment tools used in research, including food records, food diaries, 24-h recalls, food frequency questionnaires (quantitative and qualitative), and screening tools developed for specific populations/settings [88]. One of the most noted limitations of all these methods is the subjective reporting of intake by the subject or the subject’s parents, which introduces the risk of bias and reporting error [89]. The most frequently used method to measure bias or reporting error is the use of doubly labeled water to compare reported with measured energy intake [89]. More recent developments in intake assessment methodologies use approaches based on smartphone-based recordings, using manual input or photos as well as the use of AI to correct/predict food consumption [90,91]. Overall, in addition to measuring energy intake, biomarkers for the select nutrient consumption of metabolites are a suggested tool to allow for the attenuation of food intake reported with “error estimates”. Over the past decade, many biomarkers of intake have been proposed and used; a comprehensive overview is provided in a recent publication focusing on the need for individualized food intake assessment in the framework of precision nutrition [92]. As this systematic review shows, the heterogeneity of dietary intake assessment tools poses a severe limitation to the ability to summarize results across studies. Future pediatric intervention studies should be designed to use both self-reported (subjective) intake assessment tools as well as objective biomarkers to offer ways to validate the reported intake [93]. Furthermore, smaller studies might benefit from the use of 24-h recalls, considered the gold standard of intake assessment [94], while larger studies might deploy validated quantitative food frequency questionnaires [95].

Studies reporting results on the relationship between diet and blood pressure found that overall diet quality reduces SBS and DBP independently or both [18,20,32,36,37,38,41,45,46,48,51,53,54,56]. Higher diet quality was found to reduce only SBP [60] or only DPB [39,59]. A higher total energy intake and high total sugar intake increased pre-HTN, and total as well as added sugar more than doubled the risk for HTN [47]. Plant-based dietary intake reduced SBP [28] and both SBP and DBP [58). The intake of sugar increased SBP [26] while the intake of sugar-sweetened beverages had no significant effect in one study [13] but increased HTN in another [52].

Protein intake alone was also found to reduce BP, especially DBP in boys [35]. A higher intake of fruit lowered SBP and DBP and an intake of vegetables and milk reduced DBP [44]; a higher fruit and vegetable intake lowered the risk for HTN [33]. Vegetable intake alone also reduced BP [50]. Increasing the consumption of salt was associated with higher SBP [58], pre-HTN [51], and HTN [12]. Not eating breakfast increased the risk for HTN [33]. Dietary fiber lowered DBP [17] and soy intake lowered SBP [61]. Several studies found no relationship between diet and blood pressure [10,11,30,31,40,42,43,45,49,55,62,63,64,65,66,67,68].

Blood lipid levels considered risk factors for CVD, such as TG, LDL, HDL, and TC, were associated with several dietary factors. Overall, many studies found that higher diet quality was not associated with improved blood lipid levels in all children or only in some age groups [10,21,25,28,30,41,42,43,46,47,48,66,81,82,96], and others measured improved TC, LDL, and/or HDL levels [22,70,81] or only TC [15,23,45,76] or TG [31,45,46,55,72,74]. Even more surprisingly, some found a detrimental effect on HDL levels [40,60,72,74], which was attributed to a higher intake of foods increasing non-HDL lipoproteins. Others found the more expected results of higher HDL [48,55,62] and higher HDL and lower TC and LDL with increasing diet quality, especially in children and adolescents with obesity [65]. The consumption of plant-based diets and AHA guidance adherence was associated with lower TC and higher HDL [28].

Higher total dietary fiber [10,29] or fruits, vegetable, and low-sugar intake were associated with higher HDL [83] and lower LDL [25], as well as lower TG [29,60]; protein intake was also associated with lower TG [35]. One study in older children found no significant association between fiber and lipid levels in the younger children but a significant reduction in TG in high-school-age children [17]. Others found no association with changes in sugar intake and HDL [26]. Higher intakes of dietary fat lowered HDL but increased TG, TC, and LDL [73,85]. Whole milk intake was associated with higher HDL while consuming reduced-fat milk was associated with higher TG and LDL in younger children, but there was no significant effect in older children [75]. There was no significant relationship with sugar-sweetened beverages [30]. UPF and the intake of take-out food was associated with higher TG and LDL [69,71] while eating breakfast lowered TC and LDL [11]. One study examining the effect of curcumin found a significant beneficial effect on HDL levels in the unadjusted data, but after accounting for confounding factors, the results were not statistically significant [27]. Only one research team focused on fish intake and saw the expected increase in HDL levels [80]. Two studies reported results on the consumption of UPF, and both discovered that an increased consumption was associated with lower LDL and higher HDL [54,77]. The intake of diets characterized as “sweet” and “processed” was associated with higher TC and higher HDL, but the characterization of “processed” was not based on the UPF categories used in the two latter studies. There was no association between consuming breakfast and blood lipid levels in older children [26].

The studies summarized here include research in nationally representative cross-sectional studies, cohort studies, and intervention trials. The only dietary components that were consistently associated with a reduction in CVD risk factors were the Mediterranean diet, level of dietary fiber, and level of protein in the diet. Only a few studies investigated the effect of salt intake and found that salt increased SBP but not necessarily significantly and in all age groups. A comparison of the effect of diet on different age groups showed a lack of consistent results and the comparison of similar/same diet on different age groups was also not consistent. Also, many study reports did not provide information on the population being studied, such as race, ethnicity, cultural background, developmental stages, regional differences, and many more. Thus, a discussion of those contextual variables was not possible. Additionally, the inclusion criteria specified for the population of interest to be defined solely by age (children) allowed for a broad range and inclusion of different regional and global populations. This is crucial for understanding the global disease burden and risk development, as CVD mortality has trended upwards in the past 30 years [97]. The importance of understanding and addressing modifiable risk factors established through years of scientific discovery in various populations is paramount; however, because dietary norms and standards vary substantially across the globe and by local geographical regions, the generalizability of results and the efforts to evaluate food consumption patterns are limited [97]. Many studies included in this review used FFQs and indices validated for the specific region or, even more specifically, the population under study in order to address the unique food environment and societal factors. Thus, the relationship between dietary patterns and CVD risk factors provides unique guidance and clinical prevention measures but is meaningless for other regions or populations. Charchar et al. address this issue during their examination of salt consumption and blood pressure regulation [98]. The main contributors to increased salt consumption vary depending on access and dietary norms in the specific location; salt consumption can be a direct result of the availability of ultra-processed/highly palatable foods, compared to salt added during food preparation or at the table [98]. Therefore, the author’s results focus on the recommendation of reducing the consumption of ultra-processed foods, which may not lead to an overall reduction in salt intake or blood pressure in other populations that have several sources of dietary salt. Using the example of this study, the context of the study, such as local region and socio-economic factors, can lead to severe limitations due to contextual confounders. Thus, it is important to consider the unique population and dietary practices in the interpretation, especially in the decision to generalize findings to other children. While it has been established that certain aspects of diet are crucial to CVD risk factors [2], it is essential that research reports describe in detail the contextual factors and indicate universal factors so that caregivers and clinicians can use the evidence to guide children toward practices and choices that can improve health. Given the limited details provided in many of the papers included in the review about contextual factors, such as geographical region and the associated dietary practices and norms, SES, and food environment, it is not possible to generalize and transfer results to other populations for the development of specific guidelines.

Another major limitation in the interpretation of the results of this study is the very large variation in study methods used. Dietary intake assessment was self-reported (recalls, food frequency questionnaires, 24-h recalls, observations, measurements) and outcome variables were measured using different methodologies and cut points. Also, diet quality was estimated using a number of different methods, in part based on local guidelines that cannot be applied to populations living in other areas. In short, future research using standards of measurements, such as dietary indices and age-adjusted risk thresholds, would greatly improve the understanding of the relationship between diet and CVD risk. However, until internationally agreed-upon assessment tools have been developed, other methods used to summarize across varying types of exposure and outcome measures should be developed to mine the rich literature currently available. This summary of results may serve for further exploration and development of hypotheses, which could be tested through controlled trials or biomarker-based dietary assessments to strengthen the conclusions regarding dietary efficacy and potential resistance mechanisms.

## 5. Conclusions

A relatively large number of studies have investigated the relationship between diet and CVD risk factors in children; however, large heterogeneity in study methods, dietary factors examined, and outcome variables measured do not support the development of a consensus of the findings. Future research on this topic should be based on standardized methodologies for diet assessment and outcome measurement. Also, agreement on cut points to define risk factors should be generated, so research conducted in very different geographical regions in the world can inform the development of guidelines and policies to improve child nutrition, thereby lowering the risk for CVD during childhood and throughout the lifecycle.

## Figures and Tables

**Table 1:** Syntax for each of the databases.

Database Searched	Search Terms
PubMed Central 20 November 2024	(Diet [Mesh] AND “Cardiovascular Diseases” [Mesh] AND “Risk Factors” [Mesh] AND (Adolescent [Mesh] OR Children [Mesh]))
Web of Science 20 December 2024	ALL = (children OR adolescent) AND ALL = (“cardiovascular diseases”) AND ALL = (diet) AND ALL = (“risk factors”)
Embase 20 November 2024	(‘cardiovascular disease’/exp OR ‘cardiovascular disease’) AND (‘adolescence’/exp OR ‘adolescence’) AND (‘diet’/exp OR ‘diet’) AND (‘risk factors’/exp OR ‘risk factors’)

## Data Availability

No new data were created or analyzed in this study. Data sharing does not apply to this article.

## Supplementary Materials

The following supporting information can be downloaded at: https://www.mdpi.com/article/10.3390/nu18010166/s1, Table S1: Summary of publications included. Table S2: Risk of bias assessment summary.

## Abbreviations

The following abbreviations are used in this manuscript:

CVD	Cardiovascular Disease
CM	Cardiometabolic
BP	Blood Pressure
HDL	High-Density lipoprotein
LDL	Low-Density lipoprotein
TC	Total Cholesterol
TG	Triglyceride
HTN	Hypertension
UPFs	Ultra-Processed Foods
