# Supplementary material for: The Relationship Between Children’s Diet and Risk Factors for Cardiovascular Disease
Source: Nutrients. 2026 Jan 4;18(1):166. doi: 10.3390/nu18010166 (PMC12787615; doi:10.3390/nu18010166)
Supplement: Supplementary file 1 [file nutrients-18-00166-s001.zip › nutrients-3987988-supplementary.pdf]

Table S1. Summary of publications included

	1st Author	Year	Population Grade	Outcome Variables	Diet Factor of Interest	Main Findings
Muscular fitness, adherence to the Southern European Atlantic Diet and Cardiometabolic risk factors in adolescents	Agostinis-Sobrinho	2017	High	BP, TC, HDL, TG	Southern European Atlantic diet adherence	SEADiet could reduce clustered CMR in youth
Optimal Adherence to a Mediterranean Diet May Not Overcome the Deleterious Effects of Low Physical Fitness on Cardiovascular Disease Risk in Adolescents: A Cross-Sectional Pooled Analysis	Agostinis-Sobrinho	2018	Middle High	BP, TG, HDL	Mediterranean diet	No effect of diet
Interactions between genetic variants and dietary lipid composition: effects on circulating LDL cholesterol in children	Ahola-Olli	2014	Elementary	Serum lipids	Dietary lipid composition (unsaturated vs saturated fats)	PARK2 genotype variants might have an interaction with dietary fat quality on serum LDL, TC concentration
Dietary Quality Evidenced by the Healthy Eating Index and Cardiovascular Disease Risk Factors in Kuwaiti Schoolchildren	Al-Farhan	2024	Elementary	BP, calculated LDL, TC, HDL, TG	Healthy Diet Index	Sample had low diet quality, only weak associations between poor scores and elevated BP

Diet Quality Scores and Cardiometabolic Risk Factors in Mexican Children and Adolescents: A Longitudinal Analysis	Aljahdali	2022	Elementary Middle High	lipid profile and BP	DASH diet, Mediterranean Diet, and children's dietary inflammatory index	Higher diet quality associated with a better DM profile (higher DASH and MedDiet and lower C-DII scores)
Waist-to-height ratio and skipping breakfast are predictive factors for high BP in adolescents	Aparicio-Cercós	2020	Elementary Middle High	BP	Mediterranean diet quality	Skipping breakfast associated with high BP
Identification of a dietary pattern associated with greater cardiometabolic risk in adolescence	Appannah	2015	Middle High	TG, HDL, LDL	Energy dense, high fat, and low fiber diet pattern	"Energy-dense, high-fat, low-fiber" diet pattern increased CMR factors
Prevalence and determinants of hypertension and pre-hypertension among urban adolescent school students of the age group 13-17 years – A pilot study	Autkar Pusdekar	2022	Middle High	BP	Dietary practices (vegetarian, eggetarian, & mixed), added salt consumption, and frequency of junk food consumption	Risk of high BP associated with added salt and junk food more than 3x/week
Hypertension and its determinants among school going adolescents in selected urban slums of Nagpur city, Maharashtra: A cross-sectional study	Banerjee	2021	Elementary, Middle, High	BP	Dietary behavior patterns (both healthy and unhealthy)	SBP and DBP higher when consuming inadequate amounts of fruits and vegetables

The contribution of dietary composition over 25 years to cardiovascular risk factors in childhood and adulthood: the Princeton Lipid Research Study	Beck	2024	Elementary Middle High	BP and lipids	DASH diet score	No relationship between DASH adherence CVD, but saturated fat associated with components of disease risk
Dietary Patterns and Cardiovascular Risk Factors in Spanish Adolescents: A Cross-Sectional Analysis of the SI! Program for Health Promotion in Secondary Schools	Bodega	2019	Middle	BP, lipid profile	Healthy, processed, and traditional diet pattern clusters	Not a clinically relevant link between dietary patterns and CVD risk factors
Prospective association between a Mediterranean-style dietary score in childhood and cardiometabolic risk in young adults from the ALSPAC birth cohort	Buckland	2022	Elementary Middle High	TG, BP, LDL, and HDL	Mediterranean style diet score (C-rMED)	Higher Mediterranean-style diet scores in early adolescence associated healthier CMR profile
The inflammatory potential of the diet in childhood is associated with cardiometabolic risk in adolescence/young adulthood in the ALSPAC birth cohort	Buckland	2022	Elementary Middle High	HDL, LDL, TG, and BP	Child dietary inflammatory score	Pro-inflammatory diet during childhood associated with unhealthy CM profile in late adolescence and early adulthood
Childhood dietary patterns and	Bull	2016	Elementary Middle	BP, lipids, LDL	Healthy, Processed, and	No consistent evidence to support

cardiovascular risk factors in adolescence: results from the Avon Longitudinal Study of Parents and Children (ALSPAC) cohort					packed lunch dietary pattern clusters	the association of CVD risk and childhood dietary patterns
High cardiometabolic risk in healthy Chilean adolescents: associations with anthropometric, biological and lifestyle factors	Burrows	2016	High	BP, TG, TC, HDL	Food quality intake	No effect of diet
Adolescent Obesity and Its Association with Diet Quality and Cardiovascular Risk Factors	Çağiran-Yilmaz	2019	Elementary Middle High	BP, TG, TC, LDL, and HDL	Mediterranean diet	Overweight or obesity strongly associated with CVD risk, Mediterranean diet can attenuate risk factors
Association of Cardiovascular Risk Factors between Hispanic/Latino Parents and Youth: The Hispanic Community Health Study/Study of Latino Youth	Carnethon	2017	Elementary Middle High	BP, TG, TC, and LDL	Alternative Healthy Eating Index Score	Diet during youth associated with high risk for CVD as adults
Effect of multi-component school-based program on body mass index, cardiovascular and	Costa-Urrutia	2019	Elementary Middle	Lipid profile	School meals and health education	Multi-factorial intervention (physical activity, school meals, health education, and parent involvement)

diabetes risks in a multi-ethnic study						may be beneficial, but effects vary in ethnic groups
Obesogenic Behaviors and Depressive Symptoms' Influence on Cardiometabolic Risk Factors in American Indian Children	Dennison	2019	Elementary Middle	BP, lipids	Fruit and vegetable intake and sugar sweetened beverage consumption vs guidelines	Youth did not meet the dietary guidelines, diet obesogenic behavior
Alarming trends in ideal cardiovascular health among children and adolescents in Beijing, China, 2004 to 2014	Dong	2017	Elementary Middle High	TC and BP	Healthy Diet: bean curd or soybean products daily, fruits and vegetables daily, fish or fish products weekly, sugar sweetened beverages <1 time per week, salty snacks <1 time per day	Adverse trends in health behaviors (including diet) from 2004 to 2014
Longitudinal associations of away-from-home eating, snacking, screen time, and physical activity behaviors with cardiometabolic risk factors among Chinese children and their parents	Dong	2017	Elementary Middle High	BP	Away from home eating and snacking	More away-from-home meals and less fruit and vegetable intake increase risk

Takeaway meal consumption and risk markers for coronary heart disease, type 2 diabetes and obesity in children aged 9–10 years: a cross-sectional study	Donin	2018	Elementary	TC, HDL, LDL, TG, BP	To-go meals	Frequent To-Go meals associated with CVD risk
Dietary quality indices in relation to cardiometabolic risk among Finnish children aged 6-8years-The PANIC study	Eloranta	2016	Elementary	BP, TG HDL	DASH score, Baltic Sea Diet score, mediterranean diet score, and Finnish Children Healthy Eating Index	Higher FCHEI scores associated with lower CM risk in boys
Salt intake and BP in Iranian children and adolescents: a population-based study	Emamian	2021	Elementary<br>Middle<br>High	BP	Salt intake	Salt intake was 2x WHO recommendation; associated with BP
Association of whole-grain and dietary fiber intake with cardiometabolic risk in children and adolescents	Fulgoni	2020	Elementary<br>Middle<br>High	BP, HDL, and TG	Whole grains and dietary fiber intake	Dietary fiber inversely associated with several markers of CVD risk
Healthy Lifestyle Intervention and Weight Loss Improve Cardiovascular Dysfunction in Children with Obesity	Genoni	2021	Elementary<br>Middle<br>High	BP, HDL, LDL, TG, and TC	Mediterranean diet	A healthy lifestyle intervention can partially reverse cardiovascular impairment

Influence of the Mediterranean diet on carotid intima-media thickness in hypercholesterolaemic children: A 12-month intervention study	Giannini	2014	Elementary	lipid profile	Dietary intake practices	Children with hypercholesterolemia have lower LDL and cIMT after Mediterranean diet
Dietary Habits and Cardiometabolic Health in Obese Children	Gilardini	2015	Elementary<br>Middle<br>High	BP and lipids	Dietary intake patterns	Hypertensive children consume less vegetable protein; CM health improves with veggie protein and whole grain intake
Association of childhood food consumption and dietary pattern with cardiometabolic risk factors and metabolomics in late adolescence: prospective evidence from 'Children of 1997' birth cohort	He	2024	Elementary<br>Middle<br>High	BP, HDL, LDL, and TG	Dietary intake patterns in childhood	Higher consumption of fruits, vegetables, fish, and lower ice cream and sugar-sweetened beverage intake associated with lower CMR
Cardiovascular Risk Factors in Young Adolescents: Results from the National Health and Nutrition Examination Survey 1988-2016	Hecht	2021	Middle, High	TC, BP	Adherence to dietary recommendations	Modern adolescents have more dietary CV risk factors
Adolescent Diet Quality, Cardiometabolic Risk,	Hu	2023	Elementary<br>Middle<br>High	mean arterial BP, HDL, and TG	Healthy eating index scores	Adolescents with lower adherence to dietary guidelines

and Adiposity: A Prospective Cohort						and greater CMR at baseline maintained these throughout the study
Associations between Sugar Intake from Different Food Sources and Adiposity or Cardio-Metabolic Risk in Childhood and Adolescence: The Korean Child–Adolescent Cohort Study	Hur	2015	Elementary<br>Middle	TG, mean arterial pressure, and HDL	Total sugar intake and specific sugar intake	Differences in consumption of sugars from fruit and sugar-sweetened beverages affect metabolic disease
Cardiovascular risk factors for hypertension and diabetes among overweight and obese adolescents in the city of Kerbala, Iraq	Hussein Bdair	2020	Elementary<br>Middle<br>High	TG, BP, HDL, LDL, TC	Mediterranean diet quality index (KidMed)	Overweight and obesity strongly associated with multiple CVD risk factors. Med diet improves CV risk factors in adolescents
Ideal cardiovascular health at age 5–6 years and cardiometabolic outcomes in preadolescence	Jaspers Faijer-Westerink	2021	Elementary	TC, BP	Diet ideal health behaviors	Healthy diet least prevalent part of ideal CV health metric; full score improved predictions of some CVD factors
Association of heart rate and BP among European adolescents with usual food consumption: The HELENA study	Julian-Almarcegui	2016	Middle<br>High	BP	Usual intake of food	Small inverse associations between fish and dairy consumption and BP

Diet quality at age 5–6 and cardiovascular outcomes in preadolescents	Krijger	2021	Elementary Middle	BP	Dash diet score and child diet quality score	Higher diet quality in childhood (5-6 year olds) predicted better health on some CVD outcomes for pre-adolescents
Associations between Dairy Intake, Body Composition, and Cardiometabolic Risk Factors in Spanish Schoolchildren: The Cuenca Study	Lahoz-Garcia	2019	Elementary Middle	TG, lipoproteins	Dairy Intake	Normal TG and HDL levels in whole milk drinkers who are not at risk for CVD; consumers of reduced fat milk had higher levels of TG, HDL, and LDL
Dietary patterns and their association with body composition and cardiometabolic markers in children and adolescents: Genobox cohort	Latorre-Millan	2020	Elementary Middle High	BP, TG, HDL, LDL	Health-conscious vs sweet /processed	Varied results; showed that some "health-conscious" diets increase LDL and TG while sweets/processed foods reduced them; healthier overall CM profile with HC diet
Sugar-containing beverage intake at the age of 1 year and cardiometabolic health at the age of 6 years: the Generation R Study	Leermakers	2015	Elementary	HDL, BP, TG	Measured consumption of sugar beverages	Association between high sugar beverage intake at 13 months and higher CMR factors at age 6 in boys; TG, HDL, and BP most influenced
Achievement of the Targets of the 20-Year Infancy-Onset Dietary Intervention—	Lehtovirta	2021	Elementary Middle High	TG, TC	Fat quality and cholesterol intake	Meeting at least one dietary target led to lower circulating and smaller LDL

Association with Metabolic Profile from Childhood to Adulthood						
SNAP Participation and Diet-Sensitive Cardiometabolic Risk Factors in Adolescents	Leung	2017	Middle<br>High	systolic BP, TC, HDL, TG	Alternate Healthy Eating Index	Adolescents enrolled in SNAP have a higher CVD risk and lower diet quality
Plant-Based No Added Fat or American Heart Association Diets, Impact on Cardiovascular Risk in Obese Hypercholesterolemic Children and Their Parents	Mackin	2015	Elementary<br>Middle<br>High	SBP, TC, LDL	American heart association diet or plant based (no added fat) diet with nutrition education	Plant-based and AHA diets possibly beneficial for CVD risk factors
Consumption of ultra-processed foods and cardiometabolic risk factors in Brazilian adolescents: results from ERICA	Madalosso	2023	Middle<br>High	TG, HDL, LDL, TC	Ultra processed food consumption	High intake levels of ultra-processed food associated with elevated LDL levels
Lipid and saturated fatty acids intake and cardiovascular risk factors of obese children and adolescents	Maffeis	2021	Elementary<br>Middle<br>High	TG, HDL	Lipid and saturated fat intake	High saturated fat intake associated with TG and HDL levels, independent of other CV risk factors
Lifestyle and awareness of cholesterol blood levels among 29159	Martino	2019	Elementary<br>Middle	TC	Mediterranean diet	Lack of awareness of TC levels and low adherence to Mediterranean; no tendency for lower

community school children in Italy						CVD risk unless high awareness
Hypertension and obesity in Italian school children: The role of diet, lifestyle and family history	Menghetti	2015	Elementary<br>Middle<br>High	BP	Breakfast consumption and frequency of specific foods	HTN more prevalent in northern Italy due to high sodium in the diet
The association of dietary patterns and adherence to WHO healthy diet with metabolic syndrome in children and adolescents: Tehran lipid and glucose study	Mirmiran	2019	Elementary<br>Middle<br>High	BP, HDL, TG	WHO healthy diet	Unhealthy dietary pattern associated with increase in Metabolic syndrome
Prevalence of sustained hypertension and obesity among urban and rural adolescents: A school-based, cross-sectional study in North India	Mohan	2019	Elementary<br>Middle<br>High	BP	Added Salt intake	Higher salt intake increased risk for HTN
Adolescent Dietary Intakes Predict Cardiometabolic Risk Clustering	Moore	2016	Elementary<br>Middle<br>High	BP, HDL, LDL, TG	Intakes of dairy, fruit, and non-starchy vegetables	Direct effect of diet on BP, HDL, LDL, TG; girls with higher intakes of dairy, fruit, non-starchy vegetables, and grains had less accumulated risk by late adolescence

Nutritional and physical fitness parameters in adolescence impact cardiovascular health in adulthood	Morcel	2024	Middle High	HDL, non-HDL TC, BP, TC	Diet Quality index and nutritional knowledge	Ultra-processed food consumption increases risk
The association between dietary intake and cardiometabolic risk factors among obese adolescents in Indonesia	Murni	2022	High	TC, BP, lipid panel	Usual dietary intake	Significant correlation between fiber intake and HDL
The Association of Breakfast Frequency and Cardiovascular Disease (CVD) Risk Factors among Adolescents in Malaysia	Mustafa	2019	Middle	HDL, LDL, TG, TC, BP	Types of breakfast food and timing	Daily breakfast associated with lower TC and LDL
Association between Cardiovascular Risk in Adolescents and Daily Consumption of Soft Drinks: a Brazilian National Study	Neves	2022	Middle High	BP, lipids	Soft drink consumption	Daily consumption of soft drinks increased CVD risk
Dietary intake practices associated with cardiovascular risk in urban and rural Ecuadorian adolescents: a cross-sectional study	Ochoa-Avilés	2014	Middle High	BP, lipids	Grouped dietary patterns	Various dietary patterns led to high CVD risk

Added and free sugars intake and metabolic biomarkers in Japanese adolescents	Okuda	2020	Middle	BP, lipids	Intake of sugar	Added or free sugar intake associated with CVD risk
Adherence to the Japanese Food Guide: The Association between Three Scoring Systems and Cardiometabolic Risks in Japanese Adolescents	Okuda	2021	Middle	BP, lipids	Japanese Food Guide Spinning Top	Adolescents who adhere to healthy diet have low CVD risk
Interplay of the Mediterranean diet and genetic hypertension risk on BP in European adolescents: Findings from the HELENA study	Perez-Gimeno	2024	Middle, High	BP	Adherence to mediterranean diet	Higher adherence to Mediterranean diet associated with reduced BP and genetic-diet interaction influences BP in adolescents
Sociodemographic Correlates of High Cardiovascular Health Across Childhood and Adolescence: A Prospective Study Among 2 Cohorts in the ECHO Consortium	Perng	2024	Elementary Middle High	BP, non-HDL TC	Healthy Eating Index-2015	Intervention is needed in earlier teen years to prevent development of high CVD risk
Pre- and Perinatal Correlates of Ideal Cardiovascular Health (ICVH) During Early Childhood: A	Perng	2021	Elementary	TC, BP	Healthy diet (AHA)	ICVH low at age 4-7 with diet and PA being limiting factors, especially in boys

Prospective Analysis in the Healthy Start Study						
Obesity, Non-Communicable Disease (NCD) Risk Factors and Dietary Factors among Chinese School-aged Children	Piernas	2016	Elementary<br>Middle	BP, TC, LDL, HDL, TG	Dietary patterns regarding macro and micronutrients	CMR prevalent in urban and rural areas and added sugar significant increases risk
Dietary intake, obesity, and metabolic risk factors among children and adolescents in the SEACO-CH20 cross-sectional study	Ramadas	2024	Elementary<br>Middle<br>High	TC, TG, HDL, LDL	Malaysian Dietary Guidelines	Poor adherence to dietary guidelines, high levels of obesity, and increased fruit consumption linked to abdominal obesity and CVD risk
Consumption of ultra-processed food products and its effects on children's lipid profiles: A longitudinal study	Rauber	2015	Elementary	lipid profile	Processed food intake	Ultra-processed product intake increases CVD risk
Randomized 20-year infancy-onset dietary intervention, life-long cardiovascular risk factors and retinal microvasculature	Repo	2024	Elementary<br>Middle<br>High	BP, TC, HDL, TG	Heart healthy diet intervention	Lifelong cumulative CV risk factors independently associated with risk
Association between diet quality index and cardiometabolic risk factors in adolescents: Study of	Ritter	2021	Middle<br>High	TC, HDL, LDL, TG	Brazilian diet quality index	Sex affects effects of diet quality on CVD risk

Cardiovascular Risks in Adolescents (ERICA)						
From adolescence to adulthood: Mediterranean diet adherence and cardiometabolic health in a prospective cohort study	Saber	2024	Middle<br>High	lipid profile	Mediterranean diet	Greater adolescent adherence to the Mediterranean diet associated with lower risk of high TC
Childhood beverage intake and risk of hypertension and hyperlipidemia in young adults	Sakaki	2022	Elementary<br>Middle<br>High	BP, lipids	Beverage consumption (sweetened, fruit, diet soda)	Sugar-sweetened beverages in adolescence positively associated with risk of hypertension in early adulthood; fruit and diet soda no effect
Effects of curcumin on cardiovascular risk factors in obese and overweight adolescent girls: a randomized clinical trial	Saraf-Bank	2019	Middle<br>High	BP, lipid profile	500mg tumeric extract intervention	Diet and PA intervention increased HDL, improved TG/HDL ratio
Unhealthy snack intake modifies the association between screen-based sedentary time and metabolic syndrome in Brazilian adolescents	Schaan	2019	Middle<br>High	TG, BP, HDL	Unhealthy snacks	Increased screen time correlated to increased risk of MetS in adolescents who reported unhealthy snacking
Interaction effect of the mediterranean diet and an obesity genetic risk score on adiposity	Seral-Cortes	2020	Elementary<br>Middle<br>High	BP, TG, TC, HDL	Mediterranean diet	Obesity-related genotypes could impact relationship between

and metabolic syndrome in adolescents: The HELENA study						Mediterranean diet and metabolic syndrome
Dietary inflammation and cardiometabolic health in adolescents	Sethna	2021	Middle High	BP, lipid profile	Children's dietary inflammatory index	Consuming a pro-inflammatory diet in adolescence associated with increased CVD risk
Leading dietary determinants identified using machine learning techniques and a healthy diet score for changes in cardiometabolic risk factors in children: a longitudinal analysis	Shang	2020	Elementary Middle	BP, HDL, TG	identifying dietary determinants for CMR risk and developing a healthy score index	Improved healthy diet score during follow-ups associated with favorable changes in CMR scores
The Clustering of Low Diet Quality, Low Physical Fitness, and Unhealthy Sleep Pattern and Its Association with Changes in Cardiometabolic Risk Factors in Children	Shang	2020	Elementary Middle	BP, HDL, TG	Low Dietary Quality	Low diet quality results in the highest increase in CM risk factors
Diet quality and cardiometabolic health in childhood: the Generation R Study	Siddiqui	2022	Elementary	BP, TC, HDL	Diet quality score from adherence to age-specific dietary guidelines	Diet quality only significantly associated with lower BP; no effect on other risk factors

The Effects of Two Intervention Strategies to Reduce the Intake of Salt and the Sodium-To-Potassium Ratio on Cardiovascular Risk Factors. A 4-Month Randomized Controlled Study among Healthy Families	Toft	2020	Elementary<br>Middle<br>High	BP	Salt reduction strategies	Both intervention groups had no significant drop in BP
Associations between Dietary Fiber Intake in Infancy and Cardiometabolic Health at School Age: The Generation R Study	VanGijssel	2016	Elementary	TG, TC, BP	Measured dietary fiber intake	Higher fiber intake associated with higher HDL and lower TG
Mediterranean-style diet reduces metabolic syndrome components in obese children and adolescents with obesity	Velaquez-Lopez	2014	Elementary<br>Middle<br>High	lipid profile	Mediterranean-style diet	Mediterranean diet group significantly decreased TG, HDL, LDL, and TC
Protein intake in early childhood and cardiometabolic health at school age: the Generation R Study	Voortman	2016	Elementary	TG, BP, HDL, LDL, TC	High protein diets	Protein intake NOT significantly associated with BP or HDL, but with lower TG
Soy Food Intake Associated with Obesity and Hypertension in	Wang	2022	Elementary<br>Middle<br>High	BP	Soy Intake	Higher liquid soy food intake associated with lower prevalence of HTN

Children and Adolescents in Guangzhou, Southern China						
How do short-term associations between diet quality and metabolic risk vary with age?	Winpenny	2021	Elementary<br>Middle<br>High	BP, TG, HDL	DASH diet and fruit/vegetable intake	Short-term associations between diet quality and metabolic risk not consistent for adolescents and young adults
Dietary and activity habits associated with hypertension in Kunming school-aged children and adolescents: A multilevel analysis of the study of hypertension risks in children and adolescents	Yang	2024	Elementary<br>Middle<br>High	BP	Dietary habits	HTN in is high dietary modifications needed
Association between Vegetable Consumption and BP, Stratified by BMI, among Chinese Adolescents Aged 13–17 Years: A National Cross-Sectional Study	Yang	2018	Middle<br>High	BP	Vegetable intake	Daily vegetable intake of 3 servings associated with lower HTN risk
Trends and Clustering of Cardiovascular Health Metrics Among	Yang	2014	Middle<br>High	BP, TC	AHA healthy diet score	Proportion of adolescents achieving all seven CV health metrics low

U.S. Adolescents 1988–2010						and remained unchanged during 1988-2010
How Does Being Overweight Moderate Associations between Diet and BP in Male Adolescents?	Yap	2021	High	BP	Dietary habits and intake	In overweight individuals, high vegetable and milk with low levels of meat intake show lower odds of HTN
Association of health behaviors in life's essential 8 and hypertension in adolescents: a cross-sectional study from the NHANES database	Zhang	2024	Middle High	BP	Life's Essential 8 dietary habit	Better CVH score associated with less risk of HTN among boys and overweight/obese adolescents
Relationship Between Ultraprocessed Food Intake and Cardiovascular Health Among U.S. Adolescents: Results From the National Health and Nutrition Examination Survey 2007–2018	Zhang	2022	Middle High	BP, TC	Ultra processed food consumption	US adolescents consume about 2/3 of calories from UPF, graded inverse association between %cal from UPF and CVH score

Abbreviations used:
TC = total cholesterol
TG = triglycerides
LDL = low-density lipoproteins
HDL = high-density lipoproteins
CVD = cardiovascular disease
BP = BP
SBP = systolic BP

DBP = diastolic BP
CMR = cardiometabolic risk

Table S2: Risk of bias assessment summary

Study ID	Sequence Generation	Selective outcome reporting	Other sources of bias
**Agostinis-Sobrinho 2017**	low	low	low
**Agostinis-Sobrinho 2018**	low	low	low
**Ahola-Olli 2014**	low	low	low
**Al-Farhan 2024**	low	low	low
**Aljahdali 2022**	low	low	low
**Aparicio-Cercós 2020**	low	low	low
**Appannah 2015**	low	low	low
**AutkarPusdekar 2022**	low	low	low
**Banerjee 2021**	low	low	low
**Beck 2024**	low	low	low
**Bodega 2019**	low	low	low
**Buckland 2022**	low	low	low
**Buckland 2022**	low	low	low
**Bull 2016**	low	low	low
**Burrows 2016**	low	low	low
**ÇağiranYilmaz 2019**	low	low	low
**Carnethon 2017**	low	low	low

**Costa-Urrutia 2019**	low	low	low
**Dennison 2019**	high	high	high
**Dong 2017**	low	low	low
**Dong 2017**	low	low	low
**Donin 2018**	low	low	low
**Eloranta 2016**	low	low	low
**Emamian 2021**	low	low	low
**Fulgoni 2020**	low	low	low
**Genoni 2021**	low	low	low
**Giannini 2014**	low	low	low
**Gilardini 2015**	low	low	low
**Hazreen 2014**	low	low	low
**He 2024**	low	low	low
**Hecht 2021**	low	low	low
**Hu 2023**	low	low	low
**Hur 2015**	low	low	low
**HusseinBdair 2020**	low	low	low
**JaspersFaijer-Westerink 2021**	low	low	low
**Julian-Almarcegui 2016**	low	low	low
**Krijger 2021**	low	low	low
**Lahoz-García 2019**	low	low	low
**Latorre-Millán 2020**	low	low	low

**Leermakers 2015**	low	low	low
**Lehtovirta 2021**	low	low	low
**Leung 2017**	low	low	low
**Macknin 2015**	low	low	low
**Madalosso 2023**	low	low	low
**Maffeis 2021**	low	low	low
**Martino 2019**	low	low	low
**McKenzie 2020**	low	low	low
**Menghetti 2015**	low	low	low
**Mirmiran 2019**	low	low	low
**Mohan 2019**	low	low	low
**Moore 2016**	low	low	low
**Morcel 2024**	low	low	low
**Murni 2022**	low	low	low
**Mustafa 2019**	low	low	low
**Neves 2022**	low	low	low
**Ochoa-Avilés 2014**	low	low	low
**Okuda 2020**	low	low	low
**Okuda 2021**	low	low	low
**Pérez-Gimeno 2024**	low	low	low
**Perng 2021**	low	low	low
**Perng 2024**	low	low	low

**Piernas 2016**	low	low	low
**Ramadas 2024**	low	low	low
**Rauber 2015**	low	low	low
**Repo 2024**	low	low	low
**Ritter 2021**	low	low	low
**Saber 2024**	low	low	low
**Sakaki 2022**	low	low	low
**Saraf-Bank 2019**	low	low	low
**Schaan 2019**	low	low	low
**Seral-Cortes 2020**	low	low	low
**Sethna 2021**	low	low	low
**Shang 2020**	low	low	low
**Shang 2020**	low	low	low
**Siddiqui 2022**	low	low	low
**Toft 2020**	low	low	low
**vanGijssel 2016**	low	low	low
**Velázquez-López 2014**	low	low	low
**Voortman 2016**	low	low	low
**Wang 2022**	low	low	low
**Winpenny 2021**	low	low	low
**Yang 2014**	low	low	low
**Yang 2018**	low	low	low

**Yang 2024**	low	low	low
**Yap 2021**	low	low	low
**Zhang 2022**	low	low	low
**Zhang 2024**	low	low	low
